# RASR: Retinex-Guided Adaptive One-Step Diffusion for Low-Light Image Super-Resolution

**DOI:** 10.3390/s26185811

**Published:** 2026-09-14

**Authors:** Ziyu Yue, Junran Zhang, Zhixun Su

**Affiliations:** School of Mathematical Sciences, Dalian University of Technology, Dalian 116023, China; 11901015@mail.dlut.edu.cn (Z.Y.); 12501019@mail.dlut.edu.cn (J.Z.)

**Keywords:** low-light image super-resolution, diffusion model, Retinex decomposition, adaptive timestep, one-step inference, teacher–student distillation

## Abstract

Low-light image super-resolution aims to recover normal-light high-resolution images from dark low-resolution observations captured by image sensors, in which illumination attenuation, sensor noise, blur, and low resolution are entangled, making it more challenging than conventional super-resolution. Diffusion-based methods perform well on real-world super-resolution but usually require costly multi-step inference; recent one-step methods either rely on a globally fixed timestep that cannot adapt to per-sample degradation, or they directly encode the dark image into the latent space, coupling illumination bias with content degradation. To address these issues, we propose RASR, a Retinex-guided adaptive one-step diffusion framework for low-light super-resolution. We first decompose the observation into reflectance and illumination, and we use the reflectance as the content carrier to align it with the normal-light prior of the pretrained model. A latent-space teacher then constructs per-sample supervision from the low/high-quality latent discrepancy, while a lightweight student predicts the noise level solely from the Retinex representation, removing the dependence on high-quality references at inference. Finally, a single velocity-field integration on Stable Diffusion 3 yields the result, updating only low-rank adapters and lightweight modules during training. Extensive experiments on the RELLISUR benchmark show that RASR overall outperforms existing low-light and mainstream super-resolution methods in PSNR, SSIM, and LPIPS, with especially prominent gains in perceptual quality, and ablation studies validate the effectiveness of each key design.

## 1. Introduction

Image sensors are the information entry point of most visual perception systems, and their imaging quality directly determines the reliability of downstream tasks. However, under low-illumination conditions such as nighttime, dim indoor environments, or backlighting, the number of photons reaching the sensor drops sharply; constrained by the pixel area and dynamic range, sensor outputs commonly exhibit insufficient brightness, prominent read-out and dark-current noise, and loss of high-frequency detail. When the acquisition end is further limited in resolution, these degradations are superimposed on spatial down-sampling, forming a compound low-light low-resolution degradation. Such degradations widely exist in sensing applications including security surveillance, automotive night vision, and mobile photography, severely limiting the effectiveness of the corresponding systems in weak-light environments.

Low-Light image Super-Resolution (LLSR) aims to recover a normal-light high-resolution image from such dark low-resolution observations, in which illumination attenuation, sensor noise, blur, and spatial down-sampling are entangled in the same degradation process. Traditional super-resolution methods [1,2,3] usually assume a known and simple degradation (e.g., bicubic down-sampling), whereas real sensor-captured low-light images exhibit more complex and spatially varying degradations, making LLSR intrinsically harder than conventional super-resolution. Recently, generative diffusion models [4,5]—especially large-scale pretrained text-to-image (T2I) models—have shown remarkable capability in modeling natural-image priors and have been widely used for real-world super-resolution. Leveraging the strong priors in pretrained models such as Stable Diffusion [5], these methods can generate high-quality, perceptually realistic results that are difficult for GAN-based methods.

**Figure 1:**
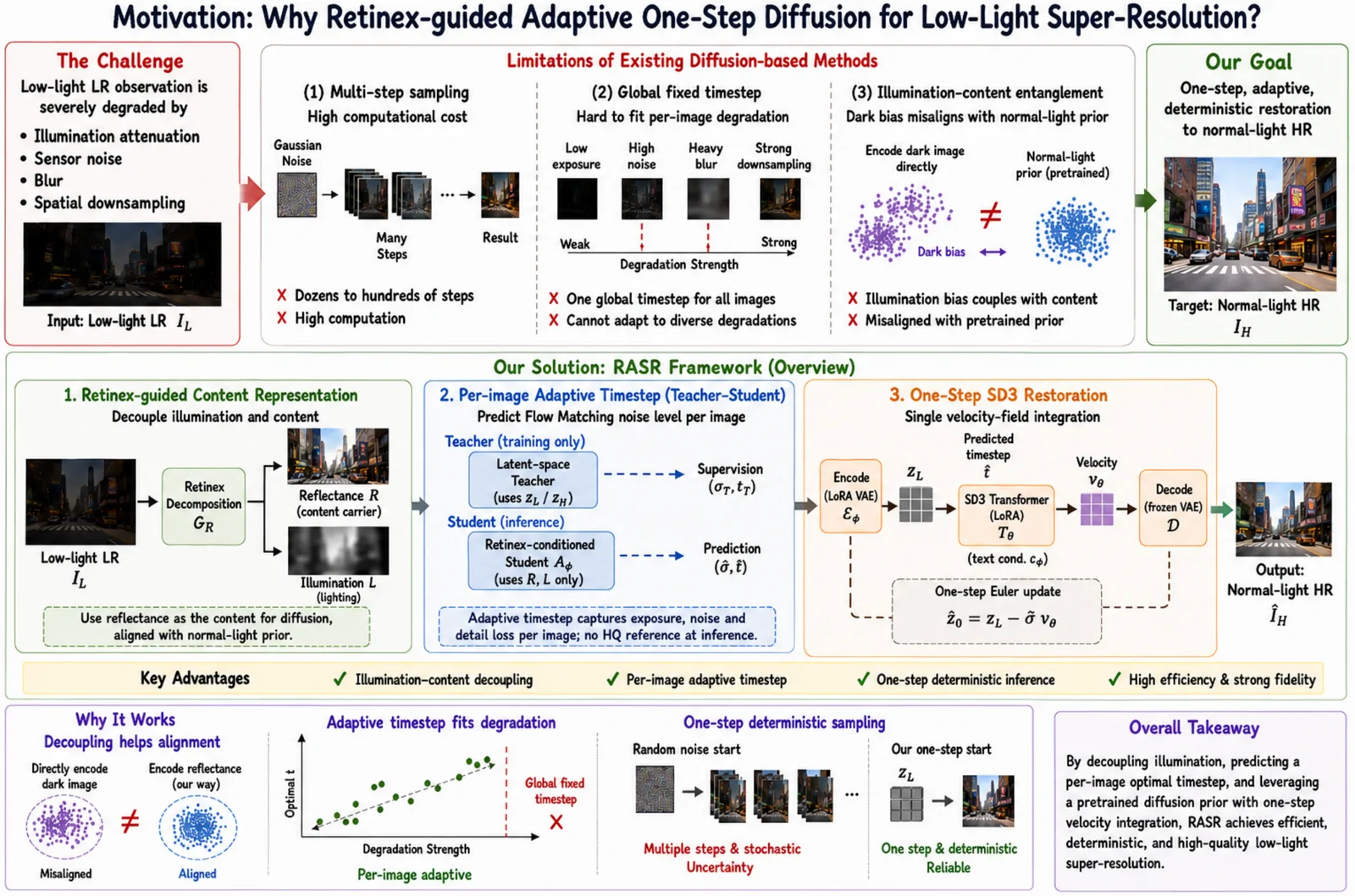
Motivation of low-light super-resolution: a globally fixed timestep cannot adapt to per-sample degradation, and directly encoding the dark image into the latent space couples illumination with content degradation.

Despite their success, existing diffusion-based super-resolution methods still have several limitations that hinder their application to low-light scenarios. First, most methods start from random Gaussian noise [6,7,8] and reconstruct high-quality images under low-quality guidance, requiring dozens or even hundreds of inference steps with huge computational cost; meanwhile, the output uncertainty introduced by random noise is unfavorable for restoration, which inherently requires deterministic and faithful reconstruction. Second, although recent one-step diffusion methods use the low-quality image as a direct starting point to accelerate inference [9,10,11], they still face two key problems under the low-light setting: on the one hand, they adopt a globally fixed timestep estimated over the whole dataset, which cannot adapt to the per-sample degradation that varies significantly across exposure, noise intensity, and texture loss; on the other hand, they directly encode the dark image into the latent space, coupling illumination bias with content degradation and breaking the alignment between the low-quality latent and the normal-light prior of the pretrained model. Figure 1 intuitively illustrates the above challenges of LLSR, which motivate our method.

To address the above problems, we propose RASR, a Retinex-guided adaptive one-step diffusion framework for low-light super-resolution. We argue that the illumination and content of a dark image should be decoupled before diffusion, and that the optimal diffusion timestep should be determined by the image itself rather than by a fixed global value. Specifically, we first decompose the observation into reflectance and illumination through a Retinex network, and we use the reflectance (rather than the original dark image) as the content carrier for diffusion restoration, aligning the latent representation with the normal-light prior of the pretrained model; then, through teacher–student distillation, we generalize the fixed timestep to a per-sample adaptive noise level, where a latent-space teacher constructs per-sample supervision from the low/high-quality latent discrepancy, and a lightweight student predicts the noise level solely from the Retinex representation, removing the dependence on high-quality references at inference; finally, a single velocity-field integration on Stable Diffusion 3 [12] yields the normal-light high-resolution result, preserving the efficiency of one-step diffusion. During training, only the low-rank adapters [13] of the VAE encoder and the SD3 Transformer, together with the lightweight Retinex and adaptive-timestep modules, are updated, while the generative backbone remains frozen.

The main contributions of this paper are summarized as follows:

- We propose RASR, a Retinex-guided adaptive one-step diffusion framework for low-light super-resolution. To the best of our knowledge, RASR is the first method that introduces the Flow Matching prior of Stable Diffusion 3 into low-light super-resolution and unifies Retinex illumination decoupling and per-sample adaptive timestep in a single-step restoration framework.
- We use the reflectance as the content carrier for diffusion restoration, decoupling illumination and content before diffusion. This alleviates the illumination–content coupling inherent in directly encoding the dark image and better aligns the latent representation with the normal-light prior of the pretrained model.
- Through latent-space teacher–student distillation, we generalize the global fixed timestep to a per-sample adaptive noise level, enabling the model to adapt to different exposure, noise, and texture degradations at inference without relying on high-quality references.
- We design a domain-consistent latent alignment together with a two-stage loss strategy that first establishes a reconstruction baseline and then gradually introduces perceptual and adversarial losses, achieving stable one-step training. Extensive experiments on the RELLISUR [14] benchmark show that RASR overall outperforms existing low-light and mainstream super-resolution methods in PSNR, SSIM, and LPIPS, and the effectiveness of each key design is validated by ablation studies.

## 2. Related Work

### 2.1. Low-Light Image Enhancement

Low-Light Image Enhancement (LLIE) aims to improve the brightness and visibility of dark images. Retinex theory [15] assumes that an image is the element-wise product of scene reflectance and ambient illumination, providing an important physical interpretation for low-light enhancement. Early deep-learning-based Retinex methods, such as RetinexNet [16], separate an image into reflectance and illumination components through a decomposition network for enhancement; KinD [17] further introduces a cross-illumination decomposition consistency constraint to alleviate the ambiguity of decomposition. More recently, Retinexformer [18] has combined Retinex decomposition with a Transformer to achieve illumination correction and detail recovery within a single stage. Beyond the Retinex paradigm, Zero-DCE [19] realizes zero-reference enhancement by estimating image-specific light-enhancement curves, EnlightenGAN [20] learns enhancement without paired supervision via global–local discriminators, SCI [21] proposes a self-calibrated illumination learning framework, and SNR-Aware [22] exploits a signal-to-noise-ratio prior to guide the fusion of Transformer and convolution. However, these methods mainly focus on illumination enhancement and ignore the loss of spatial resolution, so they cannot directly handle low-light low-resolution inputs.

### 2.2. Image Super-Resolution

Image Super-Resolution (ISR) has been dominated by deep-learning methods since SRCNN [1]. GAN-based methods represented by SRGAN [2] and ESRGAN [23] introduce perceptual loss and adversarial training, significantly improving the texture realism of generated images; Real-ESRGAN [24] and BSRGAN [25] simulate real-world degradations through high-order degradation models, advancing blind super-resolution; SwinIR [3] further enhances reconstruction with a Transformer backbone. However, the adversarial training of GANs is not sufficiently stable, and the discriminator struggles to accurately judge the quality of diverse natural images, tending to introduce unnatural artifacts in the restored results. Recently, diffusion models have achieved remarkable progress on super-resolution with their strong generative priors [6,7,8], but they usually require multi-step inference with large computational cost.

### 2.3. Diffusion Acceleration and One-Step Super-Resolution

To improve the sampling efficiency of diffusion models, various acceleration strategies have been proposed. Denoising Diffusion Probabilistic Models (DDPM) [4] and Latent Diffusion Models (LDM) [5] establish the foundation of diffusion generation; Stable Diffusion 3 [12] and Flow Matching [26,27] further model the generative process in the form of a velocity field. Consistency Models [28] and Latent Consistency Models (LCM) [29] compress multi-step sampling into few or even single steps via distillation; Adversarial Diffusion Distillation (ADD) [30] combines adversarial training to further improve the fidelity of few-step sampling. In super-resolution, StableSR [7] fine-tunes a time-aware encoder with feature warping, and ResShift [31] shortens the Markov chain via residual shifting, both achieving good results within a few steps; SeeSR [32], PASD [33], and SUPIR [34] further improve the perceptual quality of real-world super-resolution with semantic prompts or text conditions. To compress inference into a single step, SinSR [9] distills the sampling process into a student network via deterministic mapping; OSEDiff [10] directly takes the low-quality image as the diffusion starting point and applies variational score distillation; OMGSR [11] observes that the noisy latent distribution at a mid-timestep is better aligned with the low-quality latent distribution, and thus injects the low-quality latent at a precomputed mid-timestep; and TSD-SR [35] further proposes target score distillation to alleviate artifacts in one-step distillation. Although these methods achieve significant progress on real-world super-resolution, they mainly target normal-light scenarios, adopt a fixed timestep, and are not specifically designed for low-light degradation.

### 2.4. Low-Light Image Super-Resolution

Low-light image super-resolution simultaneously handles the two subtasks of illumination enhancement and spatial super-resolution. BrZoNet [36] proposes a Retinex-induced twin decoupling network that decomposes the low-light low-resolution image into reflectance and illumination maps, and it achieves simultaneous brightening and zooming through an illumination-aware interaction unit (IaIU) and a multi-stream super-resolution backbone. MSIR [37] further models joint low-light enhancement and super-resolution via multi-granularity semantic interactive representations, enabling cross-task feature interaction at different semantic levels. More recently, DARE [38] has introduced a one-step diffusion framework for low-light super-resolution, which employs degradation-aware low-rank adaptation and content-sensitive representation embedding to couple degradation priors with the reconstruction process. However, existing low-light super-resolution methods are mostly based on GAN or CNN regression frameworks, with limited generalization to complex real-world degradations; even diffusion-based DARE still directly encodes the dark observation and lacks per-sample adaptive timestep prediction. They also do not fully exploit the generative priors of large-scale pretrained diffusion models in a Retinex-decoupled, one-step manner. Building on the Retinex decoupling idea, our method incorporates low-light super-resolution into a one-step diffusion framework and introduces an adaptive timestep to accommodate per-sample degradation, thereby achieving a better balance between efficiency and quality.

## 3. Method

### 3.1. Problem Definition and Overview

Given a raw low-light low-resolution image $I_{L}^{\mathrm{raw}}\in {\left[0,1\right]}^{3\times H\times W}$ and an integer super-resolution factor r∈{2,4}, this paper aims to recover a normal-light target $I_{H}\in {\left[0,1\right]}^{3\times rH\times rW}$. Before restoration, a fixed interpolation operator $U_{r}$ maps the observation onto the target spatial grid:

(1) $${\tilde{I}}_{L}=U_{r}\left(I_{L}^{\mathrm{raw}}\right)\in {\left[0,1\right]}^{3\times rH\times rW}.$$

For conciseness, $I_{L}$ denotes ${\tilde{I}}_{L}$ hereafter. Thus, fixed interpolation performs only geometric resizing, whereas RASR learns illumination correction, latent-space restoration, and high-frequency detail reconstruction on the target grid. This task simultaneously involves illumination attenuation, sensor noise, blur, and spatial resolution loss. Directly mapping $I_{L}$ into the latent space of a pretrained diffusion model couples illumination bias with content degradation; moreover, the degradation intensity of real-world scenes varies across images, and a fixed diffusion timestep cannot adapt to different samples.

To this end, we propose the Retinex-guided Adaptive one-step diffusion Super-Resolution framework **(RASR)**. The overall method consists of three cooperating modules:

1. **Retinex-guided content representation.** The low-light observation is decomposed into reflectance and illumination, and the reflectance is used as the content carrier for diffusion restoration.
2. **Per-sample adaptive timestep.** Through teacher–student distillation, the Flow Matching noise level of each image is predicted from the Retinex representation.
3. **One-step SD3 restoration.** A single velocity-field integration is performed at the predicted timestep to generate the normal-light high-resolution result at a low sampling cost.

**Figure 2:**
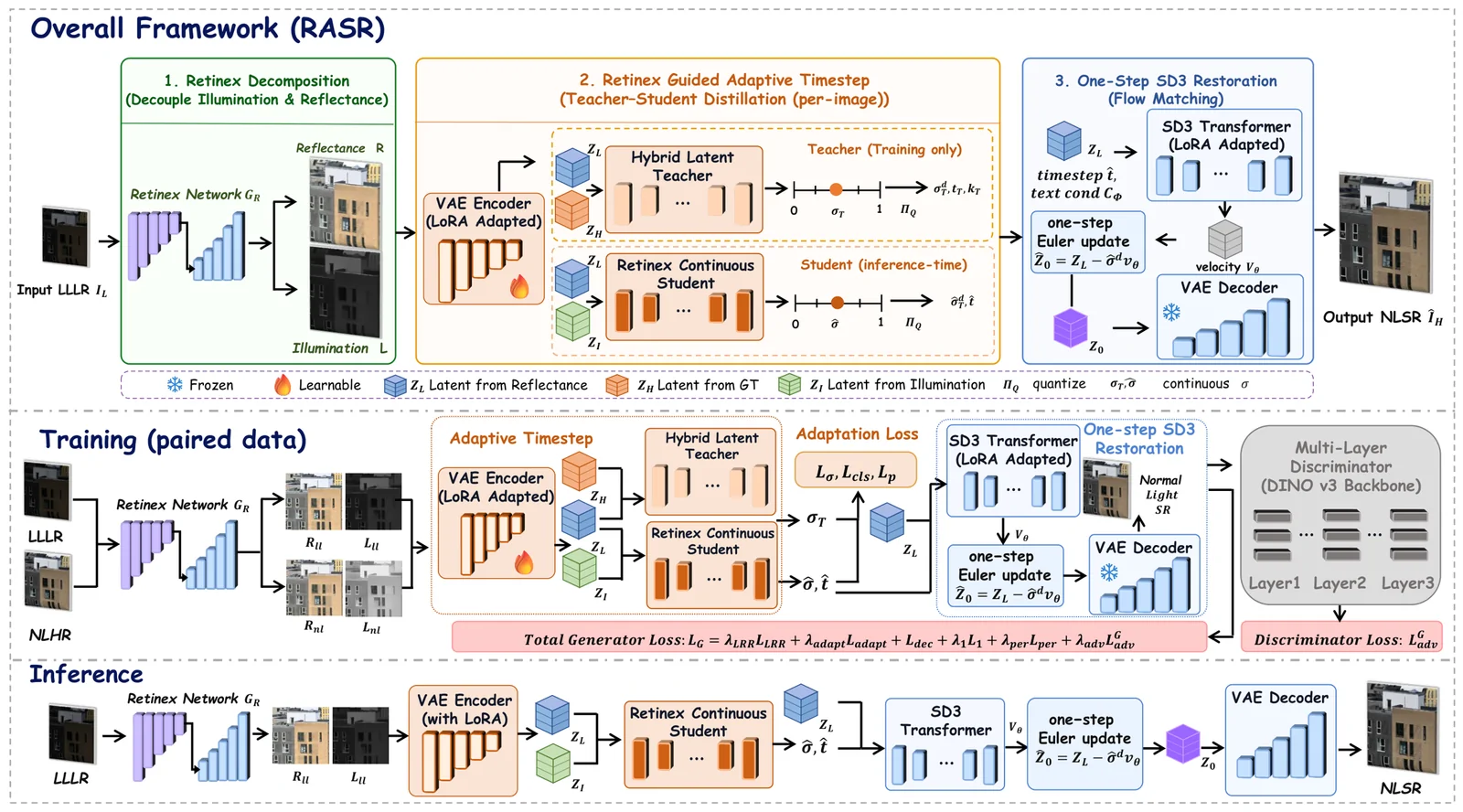
Overall framework of RASR (Retinex-guided adaptive one-step diffusion super-resolution): it comprises Retinex illumination–reflectance decoupling, per-sample adaptive timestep prediction, and one-step SD3 velocity-field integration.

Paired data $\left(I_{L}^{\mathrm{raw}},I_{H}\right)$ are used during training. After resizing and spatially aligned cropping, $I_{L}$ and $I_{H}$ have identical spatial dimensions; consequently, the Retinex outputs (R,L) share the target image grid, and the VAE latents $z_{L}$ and $z_{H}$ also have identical dimensions. This makes the reflectance-consistency, latent-alignment, pixel, perceptual, and adversarial objectives dimensionally well defined. At inference, only the low-quality branch is retained, requiring neither a high-quality reference nor a text description. The overall mapping can be written as

(2) $$I_{L}\overset{G_{R}}{\to }\left(R,L\right)\overset{A_{\psi }}{\to }\left({\hat{\sigma }}^{d},\hat{t}\right),\;{\hat{I}}_{H}=D\;\left[S_{\theta }\;\left(E_{\phi }\left(R\right),{\hat{\sigma }}^{d},\hat{t}\right)\right],$$

where $G_{R}$, $A_{\psi }$, $E_{\phi }$, $S_{\theta }$, and D denote the Retinex decomposition network, the adaptive-timestep predictor, the LoRA-equipped VAE encoder, the one-step SD3 Transformer, and the frozen VAE decoder, respectively. Figure 2 shows the overall framework of RASR.

### 3.2. Retinex-Guided Latent Representation

#### 3.2.1. Illumination–Reflectance Decoupling

According to the Retinex assumption [15], an image is the element-wise product of scene reflectance and ambient illumination:

(3) I=R⊙L,

where $R\in {\left[0,1\right]}^{3\times H\times W}$ denotes the reflectance related to object properties, and $L\in {\left[0,1\right]}^{1\times H\times W}$ denotes the spatially varying illumination. We adopt a U-Net decomposition network $G_{R}$ based on residual context blocks. The network concatenates the RGB image with its channel-wise maximum as a four-channel input, and it predicts the reflectance and single-channel illumination:

(4) $$\left(R,L\right)=G_{R}\left(I_{L}\right).$$

During training, we further decompose the normal-light image:

(5) $$\left(R_{n},L_{n}\right)=G_{R}\left(I_{H}\right),$$

and, through a cross-illumination reflectance consistency constraint $R\approx R_{n}$, we encourage the decomposition network to attribute brightness variation mainly to the illumination component while preserving geometric structure and intrinsic texture in the reflectance.

#### 3.2.2. Domain-Consistent VAE Encoding

The Retinex network operates in the image domain [0,1], whereas the input domain of the pretrained SD3 VAE is [−1,1]. We define the normalization operator

(6) $$N\left(x\right)=2x-1,\;N^{-1}\left(x\right)=\frac{x+1}{2}.$$

The low-quality latent is obtained by encoding the reflectance:

(7) $$z_{L}=s_{\mathrm{vae}}\;\left[E_{\phi }\;\left(N(R)\right)-b_{\mathrm{vae}}\right],$$

and the high-quality latent is obtained by a frozen reference encoder:

(8) $$z_{H}=s_{\mathrm{vae}}\;\left[E_{0}\;\left(N(I_{H})\right)-b_{\mathrm{vae}}\right],$$

where $s_{\mathrm{vae}}$ and $b_{\mathrm{vae}}$ are the scaling factor and shift factor of the SD3 VAE, respectively. $E_{0}$ is completely frozen, and $E_{\phi }$ is updated only through low-rank adapters. Compared with directly encoding the dark observation, the reflectance latent reduces the global illumination shift, making it easier to align with the normal-light content in the pretrained generative prior.

### 3.3. Retinex Adaptive Timestep

One-step diffusion restoration is highly sensitive to the integration starting point. OMGSR [11] selects a fixed mid-timestep via dataset-level SNR matching; we generalize this to per-sample estimation and adopt a teacher–student framework to address the inaccessibility of HQ images at inference.

#### 3.3.1. Flow Matching Schedule Mapping

Let the discrete schedule grid of SD3 be

(9) $$Q={\left\{\left(\sigma_{k},t_{k}\right)\right\}}_{k=1}^{K}.$$

For a continuous noise level σ, we define the nearest-neighbor mapping

(10) $$k^{*}=\arg\min_{k}\left|\sigma -\sigma_{k}\right|,\;\Pi_{Q}\left(\sigma \right)=\left(\sigma_{k^{*}},t_{k^{*}}\right).$$

This mapping guarantees that the predicted noise level is strictly consistent with the pretrained Flow Matching scheduler.

#### 3.3.2. Hybrid Latent-Space Teacher

The teacher uses paired latents $\left(z_{L},z_{H}\right)$ during training. All statistics below are computed per sample over the latent channels and spatial positions, with population variance and covariance. Let $\mu_{L}$ and $\mu_{H}$ denote the means. We first align the low-quality latent using

(11) $$a=\frac{\mathrm{Cov}(z_{L},z_{H})}{\max(\mathrm{Var}\left(z_{L}\right),\epsilon )},\;b=\mu_{H}-a\mu_{L},\;{\bar{z}}_{L}=az_{L}+b.$$

For the residual $e={\bar{z}}_{L}-z_{H}$, define

(12) $$m_{\mathrm{abs}}={\mathrm{median}}_{i}\left|e_{i}\right|,\;T=3\max\left(m_{\mathrm{abs}},\epsilon \right),\;\rho \left(e_{i}\right)=\mathrm{clamp}\left(e_{i},-T,T\right).$$

The energy estimation term is written as

(13) $$\sigma_{e}=\sqrt{\frac{E\left[\rho {\left({\bar{z}}_{L}-z_{H}\right)}^{2}\right]}{\nu +E\left[z_{H}^{2}\right]+\epsilon }},$$

where ρ clips residual values to the stated endpoints. We set the unit noise power ν=1 and the numerical stabilizer $\epsilon =10^{-8}$.

The energy term mainly measures the residual magnitude but cannot fully describe correlated structural changes. We therefore further standardize the latents per sample and construct a covariance estimate

(14) $$\sigma_{c}=1-\frac{\mathrm{Cov}({\tilde{z}}_{L},{\tilde{z}}_{H})}{\mathrm{Var}({\tilde{z}}_{H})+\epsilon },$$

where $\tilde{z}$ denotes the zero-mean, unit-variance latent. To explicitly characterize high-frequency degradation, we compute the structural difference using the Sobel operator:

(15) $$\sigma_{g}=\frac{E\left[\left|\nabla {\tilde{z}}_{L}-\nabla {\tilde{z}}_{H}\right|\right]}{E\left[\left|\nabla {\tilde{z}}_{H}\right|\right]+E\left[\left|\nabla {\tilde{z}}_{L}-\nabla {\tilde{z}}_{H}\right|\right]+\epsilon }.$$

The final teacher target is a weighted sum of the three terms:

(16) $$\sigma_{T}=w_{e}\sigma_{e}+w_{c}\sigma_{c}+w_{g}\sigma_{g},$$

where $\left(w_{e},w_{c},w_{g}\right)=\left(0.45,0.35,0.20\right)$. Each of the three estimates is clipped to $\left[\sigma_{\min},\sigma_{\max}\right]$ before combination; the weighted $\sigma_{T}$ is clipped again before nearest-neighbor mapping by $\Pi_{Q}$. We use the SD3 FlowMatchEulerDiscreteScheduler (diffusers 0.34.0), with set_timesteps(1000) and shift 3.0. After excluding the terminal zero, K=1000, $\sigma_{\min}\approx 8.93\times 10^{-3}$, and $\sigma_{\max}=1.0$. This yields $\left(\sigma_{T}^{d},t_{T},k_{T}\right)$ even when a raw estimate lies outside [0,1]. Both input latents are detached, and teacher construction, schedule mapping, and soft-target generation are performed under no_grad; targets are recomputed for each pair and treated as constants during the corresponding student update.

#### 3.3.3. Retinex-Conditioned Student

The teacher relies on HQ latents and cannot be used directly at real inference. We therefore design a lightweight student $A_{\psi }$ that predicts the noise level solely from (R,L).

The student contains two asymmetric encoders. The reflectance encoder adopts residual convolution blocks with efficient channel attention to preserve texture and semantic structure; the illumination encoder adopts multi-scale dilated convolutions to capture local lighting and large-range brightness variation. Let their outputs be

(17) $$F_{R},v_{R}=F_{R}\left(R\right),\;F_{L},v_{L}=F_{L}\left(L\right).$$

At the spatial level, the illumination vector produces gating, shift, and contextual modulation to conditionally fuse the reflectance features:

(18) $$F=\mathrm{SiLU}\;\left\{M\;\left[F_{R},F_{R}+g\left(v_{L}\right)⊙\delta \left(v_{L}\right),F_{R}⊙c\left(v_{L}\right)\right]+F_{R}\right\},$$

where M concatenates the three branches and fuses them using a 1×1 convolution followed by a 3×3 convolution; the residual connection preserves the original reflectance features. The fused map is then globally average-pooled as

(19) $$v_{F}=\mathrm{GAP}\left(F\right).$$

At the vector level, we additionally extract the illumination mean, standard deviation, minimum, maximum, and the 25%/75% quantiles, denoted $s_{L}$. The fusion module jointly models the illumination-conditioned content representation, illumination, multiplicative interaction, absolute difference, and explicit statistics:

(20) $$h=V\;\left[v_{F},v_{L},v_{F}⊙v_{L},\left|v_{F}-v_{L}\right|,s_{L}\right].$$

The network predicts using both regression and classification heads:

(21) $$\hat{\sigma }=\sigma_{\min}+\mathrm{sigmoid}\;\left(f_{\mathrm{reg}}\left(h\right)\right)\left(\sigma_{\max}-\sigma_{\min}\right),\;p=\mathrm{softmax}\;\left(f_{\mathrm{cls}}\left(h\right)\right).$$

By default, $\Pi_{Q}\left(\hat{\sigma }\right)$ yields the $\left({\hat{\sigma }}^{d},\hat{t}\right)$ required for the actual integration; the classification head serves as auxiliary schedule supervision.

#### 3.3.4. Distillation Objective

The regression branch adopts a Smooth-$L_{1}$ loss:

(22) $$L_{\sigma }=SmoothL1\left(\hat{\sigma },\sigma_{T}\right).$$

To reduce the abrupt supervision change at discrete grid boundaries, we construct soft labels with temperature τ from the teacher continuous noise level:

(23) $$q_{k}=\frac{\exp(-|\sigma_{T}-\sigma_{k}|/\tau )}{\sum_{j}\exp(-|\sigma_{T}-\sigma_{j}\left|/\tau \right)},$$

and define the classification loss

(24) $$L_{\mathrm{cls}}=-\sum_{k}q_{k}\log p_{k}.$$

In addition, letting ${\hat{\sigma }}_{p}=\sum_{k}p_{k}\sigma_{k}$, a consistency term constrains the expectation of the classification distribution:

(25) $$L_{p}=SmoothL1\left({\hat{\sigma }}_{p},\sigma_{T}\right).$$

The complete adaptive-timestep loss is

(26) $$L_{\mathrm{adapt}}=\lambda_{\sigma }L_{\sigma }+\lambda_{\mathrm{cls}}L_{\mathrm{cls}}+\lambda_{p}L_{p},$$

with default settings $\lambda_{\sigma }=1.0$, $\lambda_{\mathrm{cls}}=0.5$, $\lambda_{p}=0.25$, and τ=0.01.

### 3.4. Adaptive One-Step SD3 Restoration

#### 3.4.1. Latent Representation Refinement

Given the student-predicted discrete noise level ${\hat{\sigma }}^{d}$, we perturb the HQ latent according to the Flow Matching forward trajectory:

(27) $${\tilde{z}}_{H}=\left(1-{\hat{\sigma }}^{d}\right)z_{H}+{\hat{\sigma }}^{d}\epsilon ,\;\epsilon ∼N\left(0,I\right).$$

The latent representation refinement loss is defined as

(28) $$L_{\mathrm{LRR}}={∥z_{L}-{\tilde{z}}_{H}∥}_{2}^{2}.$$

This objective constrains the output of the reflectance encoder onto the latent manifold of the pretrained SD3 at the corresponding noise level so that the LQ representation can directly serve as the starting point of adaptive one-step integration.

#### 3.4.2. One-Step Velocity-Field Integration

The SD3 Transformer takes $z_{L}$, the predicted timestep $\hat{t}$, and the empty text condition $c_{⌀}$ as inputs to estimate the velocity field:

(29) $$v_{\theta }=T_{\theta }\;\left(z_{L},\hat{t},c_{⌀}\right).$$

A single explicit Euler integration then recovers the clean latent:

(30) $${\hat{z}}_{0}=z_{L}-{\hat{\sigma }}^{d}v_{\theta }.$$

The output image is

(31) $${\hat{I}}_{H}=N^{-1}\;\left\{D\;\left[\frac{{\hat{z}}_{0}}{s_{\mathrm{vae}}}+b_{\mathrm{vae}}\right]\right\}.$$

The backbone SD3 Transformer and VAE decoder remain frozen, and only low-rank adapters [13] are injected into the Transformer and the VAE encoder. This design preserves the large-scale generative prior while significantly reducing the training cost of low-light super-resolution.

### 3.5. Joint Optimization Objective

#### 3.5.1. Retinex Decomposition Constraint

The decomposition loss consists of reconstruction, illumination smoothness, illumination prior, and reflectance consistency:

(32) $$\begin{array}{cc} L_{\mathrm{dec}}= & \lambda_{ll}{∥R⊙L-I_{L}∥}_{1}+\lambda_{nl}{∥R_{n}⊙L_{n}-I_{H}∥}_{1}\\ \; & +\lambda_{\mathrm{satv}}\left[\mathrm{SATV}\left(L,I_{L}\right)+\mathrm{SATV}\left(L_{n},I_{H}\right)\right]\\ \; & +\lambda_{\max}\left[{∥L-\max_{c}\left(I_{L}\right)∥}_{2}^{2}+{∥L_{n}-\max_{c}\left(I_{H}\right)∥}_{2}^{2}\right]\\ \; & +\lambda_{R}{∥R-R_{n}∥}_{1}, \end{array}$$

where SATV constrains the illumination map to remain continuous in smooth regions while allowing structural variation at image edges.

#### 3.5.2. Pixel and Perceptual Reconstruction

The pixel reconstruction losses are defined as

(33) $$L_{1}={∥{\hat{I}}_{H}-I_{H}∥}_{1},\;L_{2}={∥{\hat{I}}_{H}-I_{H}∥}_{2}^{2}.$$

We use a modified DISTS-style objective [39] to compare channel-wise luminance and structure statistics of the RGB image and the first three frozen DINOv3-ConvNeXt-Large feature stages [40]. The similarities are aggregated using fixed channel weights:

(34) $$L_{\mathrm{per}}={\mathrm{DISTS}}_{\mathrm{DINOv}3}\;\left({\hat{I}}_{H},I_{H}\right).$$

The RGB level uses [0,1] inputs, while the deep-feature branch applies ImageNet normalization. The feature backbone is frozen, but image gradients are retained. Appendix A.4 defines the similarities, weights, and inter-stage L2 pooling. This training objective is distinct from the AlexNet LPIPS metric used for evaluation.

#### 3.5.3. Multi-Layer Adversarial Learning

The discriminator reuses the frozen DINOv3-ConvNeXt [40] backbone and attaches trainable multi-scale discriminative heads on the first three layers of features. The generator adversarial objective is

(35) $$L_{\mathrm{adv}}^{G}=E_{{\hat{I}}_{H}}\;\left[l_{\mathrm{bce}}\;\left(D({\hat{I}}_{H}),0.8\right)\right],$$

and the discriminator objective is

(36) $$L_{\mathrm{adv}}^{D}=E_{I_{H}}\;\left[l_{\mathrm{bce}}\;\left(D(I_{H}),0.8\right)\right]+E_{{\hat{I}}_{H}}\;\left[l_{\mathrm{bce}}\;\left(D({\hat{I}}_{H}),0\right)\right].$$

To balance training speed and the generator–discriminator dynamics, the generator is updated at every iteration, while the discriminator is updated once every two iterations. When computing the generator gradient, the discriminator parameters are frozen, but the gradient of the discriminative path with respect to the generated image is retained.

Here, BCE-with-logits is averaged over spatial positions and the batch and summed over the three heads; real and generator targets use 0.8, and discriminator fake targets use 0. Appendix A.3 specifies the head channels, strides, normalization, and augmentation.

#### 3.5.4. Overall Objective

The final generator objective is

(37) $$L_{G}=\lambda_{\mathrm{LRR}}L_{\mathrm{LRR}}+\lambda_{\mathrm{adapt}}L_{\mathrm{adapt}}+L_{\mathrm{dec}}+\lambda_{1}L_{1}+\lambda_{2}L_{2}+\lambda_{\mathrm{per}}\left(s\right)L_{\mathrm{per}}+\lambda_{\mathrm{adv}}\left(s\right)L_{\mathrm{adv}}^{G}.$$

where *s* denotes the training step. We set $\lambda_{\mathrm{LRR}}=5$, $\lambda_{\mathrm{adapt}}=1$, $\lambda_{1}=1$, and $\lambda_{2}=1$. Following the proposed two-stage strategy, $\left(\lambda_{\mathrm{per}}\left(s\right),\lambda_{\mathrm{adv}}\left(s\right)\right)$ is set to (1,0) for steps 0–7999, linearly transitioned toward (3,0.1) over steps 8000–9999, and kept at (3,0.1) from step 10,000 to step 11,999. The generator and discriminator are optimized independently, with the discriminator updated once every two steps after the adversarial loss is activated.

### 3.6. Training and Inference Strategy

The Retinex branch is pretrained on the 3610 RELLISUR training pairs using the decomposition objective in Section 3.5.1, without external or test data. During joint training, its parameters are frozen for the first two epochs and unfrozen at the start of the third epoch (zero-based index 2); they remain registered in the generator optimizer throughout. Initialization and data-pairing details are provided in Appendix A.1.

The training procedure can be summarized as follows:

1. $I_{L}\leftarrow U_{r}\left(I_{L}^{\mathrm{raw}}\right)$, followed by spatially aligned cropping of $\left(I_{L},I_{H}\right)$.
2. $\left(R,L,R_{n},L_{n}\right)\leftarrow \mathrm{Retinex}\left(I_{L},I_{H}\right)$.
3. $z_{L}\leftarrow {\mathrm{VAE}}_{\phi }\left(N\left(R\right)\right)$, $z_{H}\leftarrow {\mathrm{VAE}}_{0}\left(N\left(I_{H}\right)\right)$.
4. $\sigma_{T}\leftarrow \mathrm{Teacher}\left(z_{L},z_{H}\right)$.
5. $\left({\hat{\sigma }}^{d},\hat{t}\right)\leftarrow \Pi_{Q}\left(\mathrm{Student}\left(R,L\right)\right)$.
6. ${\hat{z}}_{0}\leftarrow z_{L}-{\hat{\sigma }}^{d}\;T_{\theta }\left(z_{L},\hat{t},c_{⌀}\right)$.
7. ${\hat{I}}_{H}\leftarrow N^{-1}\left({\mathrm{VAE}}_{\mathrm{Dec}}\left({\hat{z}}_{0}\right)\right)$.
8. Update the generator; update the discriminator once every two steps.

At inference, the raw input is first resized by $U_{r}$ to the requested target resolution, while the HQ encoder, teacher, perceptual network, and discriminator are removed, retaining only

(38) $$I_{L}^{\mathrm{raw}}\overset{U_{r}}{\to }I_{L}\to \left(R,L\right)\to \left({\hat{\sigma }}^{d},\hat{t}\right)\to z_{L}\to {\hat{z}}_{0}\to {\hat{I}}_{H}.$$

Therefore, RASR performs a single Flow Matching integration step rather than iterative denoising. For images that fit within one latent tile, this corresponds to one SD3 Transformer evaluation. For ultra-high-resolution images, we apply overlapping latent-space tiling with Gaussian-weighted fusion; each tile is evaluated once, and the adaptive-timestep prediction is performed on down-sampled Retinex features to control the additional computational overhead.

### 3.7. Difference from Fixed-Timestep OMGSR

Fixed-timestep OMGSR [11] estimates a single global optimal timestep on the training set and uses the same integration starting point for all samples. In contrast, our method treats the timestep as a latent variable jointly determined by content, illumination, and degradation; constructs per-sample supervision via a latent-space teacher; and distills it into a student that relies only on the Retinex representation. This design preserves the efficiency of one-step diffusion while enhancing the model’s adaptability to different degrees of exposure, noise, and texture loss.

## 4. Experiments

### 4.1. Experimental Setup

**Dataset.** We use the RELLISUR [14] real-world low-light super-resolution benchmark, which includes camera-captured observations at multiple exposure levels. The ×2 and ×4 models are each trained on 3610 paired samples from the training split and evaluated on 425 paired samples from the corresponding Test_crop split. The training and test LQ filename sets are disjoint. Appendix A.1 gives the exact paired directories and filename-matching protocol.

**Evaluation metrics.** We report the peak signal-to-noise ratio (PSNR) [41], structural similarity (SSIM) [42], and learned perceptual image patch similarity (LPIPS) [43]. Outputs are clipped to [0,1] and saved as 8-bit RGB PNGs. PSNR uses all color channels, and SSIM is computed per channel and averaged; both use scikit-image with data_range = 255, without Y-channel conversion or border cropping. LPIPS uses its official AlexNet implementation with RGB inputs normalized to [−1,1]. This evaluation backbone differs from the DINOv3 features used by the training loss. Additional evaluation details are given in Appendix A.1.

**Implementation details.** The backbone is a pretrained Stable Diffusion 3 [12]. For both scales, the raw LQ image is first resized to the corresponding native GT dimensions using fixed Lanczos interpolation; the resized LQ and GT are then subjected to the same random 1024×1024 crop and horizontal flip. The ×2 and ×4 models are trained with the ×2 and ×4 targets of RELLISUR, respectively. We inject a rank-64 LoRA into the VAE encoder and a rank-128 LoRA into the SD3 Transformer [13], and we train the Retinex decomposition network and the adaptive-timestep module. Both adapters use α=r, giving a scaling factor of 1. Appendix A.2 lists the target modules, dropout, and initialization. The learning rate is $2\times 10^{-5}$ with a cosine schedule and 1500-step warmup, the batch size is 1, and training runs for 12,000 steps with bf16 mixed precision; the discriminator is updated once every two steps. At inference, the requested scale factor determines the target grid before RASR restoration. All models are trained on a single NVIDIA A800-SXM4 GPU with 80 GB memory.

**Computational efficiency.** Table 1 reports complete restoration-forward timing on an NVIDIA A800-SXM4 80 GB GPU with BF16 precision and batch size 1. Each resolution uses one prepared input, measured ten times after two warm-up runs; the reported variation is the sample standard deviation across these repeats. The restoration network contains 2.118B parameters after LoRA merging and 179.33M trainable parameters during joint training; including discriminator heads gives 184.31M trainable parameters. Each latent tile requires one Transformer evaluation, with 4 and 16 tiles at the tested resolutions. Appendix A.5 specifies the input preparation, timing boundaries, parameter-count scope, reserved memory, and tiling settings.

### 4.2. Comparison with Existing Methods

To verify the effectiveness of our method, we compare RASR (two-stage loss) with existing mainstream super-resolution and low-light methods on the RELLISUR evaluation set. All compared methods are retrained on the same RELLISUR training set used in this work. Training configurations follow the respective official implementations. The compared methods cover six categories: (1) CNN-based regression super-resolution methods EDSR [44], D-DBPN [45], RDN [46], RCAN [47], SRFBN [48], PAN [49], and MSRResNet [23]; (2) the GAN-based super-resolution method ESRGAN [23]; (3) Transformer-based super-resolution and restoration methods SwinIR [3], Restormer [50], SRFormer [51], HAT [52], HiT-SR [53], and CATANet [54]; (4) cascaded low-light enhancement and super-resolution pipelines RUAS [55]→SwinIR and LLFormer [56]→HAT; (5) low-light enhancement and low-light super-resolution methods MIRNet [57], BrZoNet [36], MSIR [37], and DARE [38]; and (6) methods originally designed for other restoration tasks—DHGM [58] for deraining + super-resolution and DOD [59] for all-in-one image restoration. All methods are evaluated on the same 425-image RELLISUR test set under an identical PSNR/SSIM/LPIPS computation protocol. Table 2 presents the quantitative comparison on the ×2 and ×4 tasks.

From Table 2, the following conclusions can be drawn:

- **Comprehensive lead on the ×2 task.** RASR outperforms all compared methods on PSNR, SSIM, and LPIPS, where PSNR reaches 23.70 dB, an improvement of +0.91 dB over BrZoNet designed specifically for low-light super-resolution, with SSIM improved by +0.026 and LPIPS reduced by −0.041. This demonstrates the clear advantage of our method on the ×2 low-light super-resolution task.
- **Also leading on the ×4 task.** On the more severely degraded ×4 task, the LPIPS of RASR reaches 0.319, a large reduction of −0.064 over BrZoNet (0.383), indicating that the restored images are perceptually much closer to real normal-light images; meanwhile, PSNR (21.45 dB) is also slightly higher than that of BrZoNet (21.41 dB). Overall, RASR leads all compared methods on PSNR and LPIPS on the ×4 task; SSIM (0.729) is roughly comparable to DHGM [58] (0.733), a deraining + super-resolution method retrained on the same RELLISUR training set, and the perceptual-quality improvement is especially prominent.

Overall, RASR achieves competitive performance on low-light super-resolution, particularly leading in perceptual quality, which validates the effectiveness of combining Retinex-guided representation with the one-step diffusion prior.

### 4.3. Ablation Studies

To systematically verify the role of each key design in RASR, this section conducts ablation analysis on three core components: the two-stage loss strategy, the reflectance-guided content representation, and the per-sample adaptive timestep. All ablation experiments use the same dataset, optimization budget, and evaluation protocol, and they are evaluated at 12,000 training steps.

#### 4.3.1. Two-Stage Loss Strategy

To verify the effectiveness of the two-stage loss strategy, we compare the proposed schedule with a conventional single-stage configuration that keeps the perceptual and adversarial weights fixed throughout training. The proposed strategy divides training into two stages:

- **Stage 1 (reconstruction, step 0–7999)**: Dominated by reconstruction losses ($L_{1}$, $L_{2}$, $L_{\mathrm{LRR}}$), with a low perceptual weight ($\lambda_{\mathrm{per}}=1$) and the adversarial loss turned off ($\lambda_{\mathrm{adv}}=0$).
- **Stage 2 (refinement, step 8000–11,999)**: During the transition at step 8000–9999, the perceptual weight rises linearly from 1 to 3 and the adversarial weight from 0 to 0.1; after step 10,000, the final perceptual and adversarial weights are kept ($\lambda_{\mathrm{per}}=3$, $\lambda_{\mathrm{adv}}=0.1$).

The core idea of the two-stage strategy is to first establish a stable latent and pixel foundation with reconstruction losses, and then gradually introduce perceptual and adversarial losses to refine textures, thereby avoiding the discriminator dominating the optimization too early. Table 3 presents the quantitative comparison of the two strategies at 12,000 steps.

As shown in Table 3, the two-stage loss consistently outperforms the single-stage loss on both the ×2 and ×4 tasks: on ×2, PSNR improves by +0.70 dB, SSIM by +0.020, and LPIPS decreases by −0.010; on ×4, the improvements are more significant, with PSNR +0.54 dB, SSIM +0.017, and LPIPS −0.029. Notably, the ×4 task is more severely degraded and harder to reconstruct, and the design of first building a reconstruction foundation and then refining textures yields larger gains, which validates the adaptability of this strategy to difficult tasks.

#### 4.3.2. Key-Component Ablation: Reflectance Representation and Adaptive Timestep

To verify the individual contributions of the reflectance-guided representation and the per-sample adaptive timestep, we construct two controlled settings and compare them with their common baseline (reflectance + adaptive timestep):

- **Direct dark-image encoding**: The reflectance R obtained by Retinex decomposition is replaced by the original dark low-resolution image $I_{L}$ encoded directly into the VAE, with all other components and the training pipeline kept identical.
- **Fixed timestep**: The adaptive-timestep module is replaced by a fixed global mid-timestep, no longer predicting the per-sample noise level from image content, with all other components and the training pipeline kept identical. Both scales use zero-based schedule index 700, corresponding to t≈564.13 and σ≈0.5641 on the 1000-point SD3 schedule.

Table 4 presents the quantitative results of the two ablations on the ×2 and ×4 tasks.

As shown in Table 4, the baseline achieves the highest PSNR and lowest LPIPS on both scales. Both ablated components yield overall improvements in PSNR and LPIPS, while SSIM remains roughly comparable on the ×4 task:

- **Reflectance-guided representation**: Compared with direct dark-image encoding, on ×2, PSNR improves by +0.38 dB, SSIM by +0.013, and LPIPS decreases by −0.014, all three metrics being superior; on ×4, PSNR improves by +0.49 dB and LPIPS decreases by −0.019, with more prominent perceptual improvement and roughly comparable SSIM. This indicates that illumination–reflectance decoupling before diffusion effectively alleviates the coupling of illumination bias and content degradation, better aligning the latent representation with the normal-light prior of the pretrained model, with larger gains on the more severely degraded ×4 task.
- **Per-sample adaptive timestep**: Compared with the fixed setting, on ×2, PSNR improves by +0.34 dB, SSIM by +0.015, and LPIPS decreases by 0.009; on ×4, the mean changes are smaller: +0.09 dB in PSNR and a decrease of 0.004 in LPIPS, accompanied by a slight decrease in SSIM. The observed benefit differs across scales and metrics.

We further evaluate timestep prediction on all 425 test pairs. The continuous-noise-level MAE between the student and teacher is 0.0259 on ×2 and 0.0266 on ×4. At ×4, 402 of 425 mapped predictions select t≈181.58, indicating a limited timestep response. The full errors, exposure-grouped distributions, and diagnostic protocol are given in Appendix A.6.

### 4.4. Qualitative Comparison

**Figure 3:**
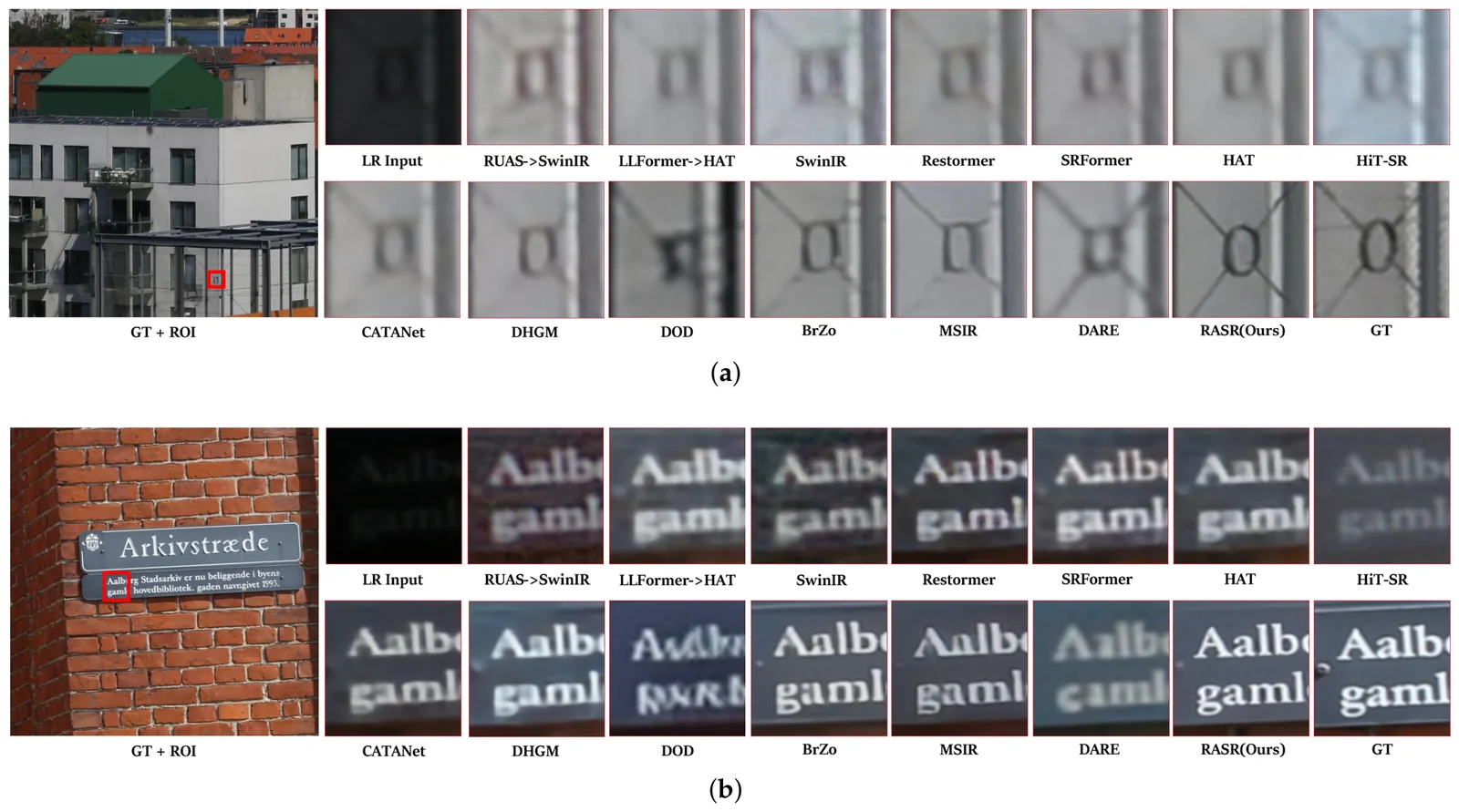

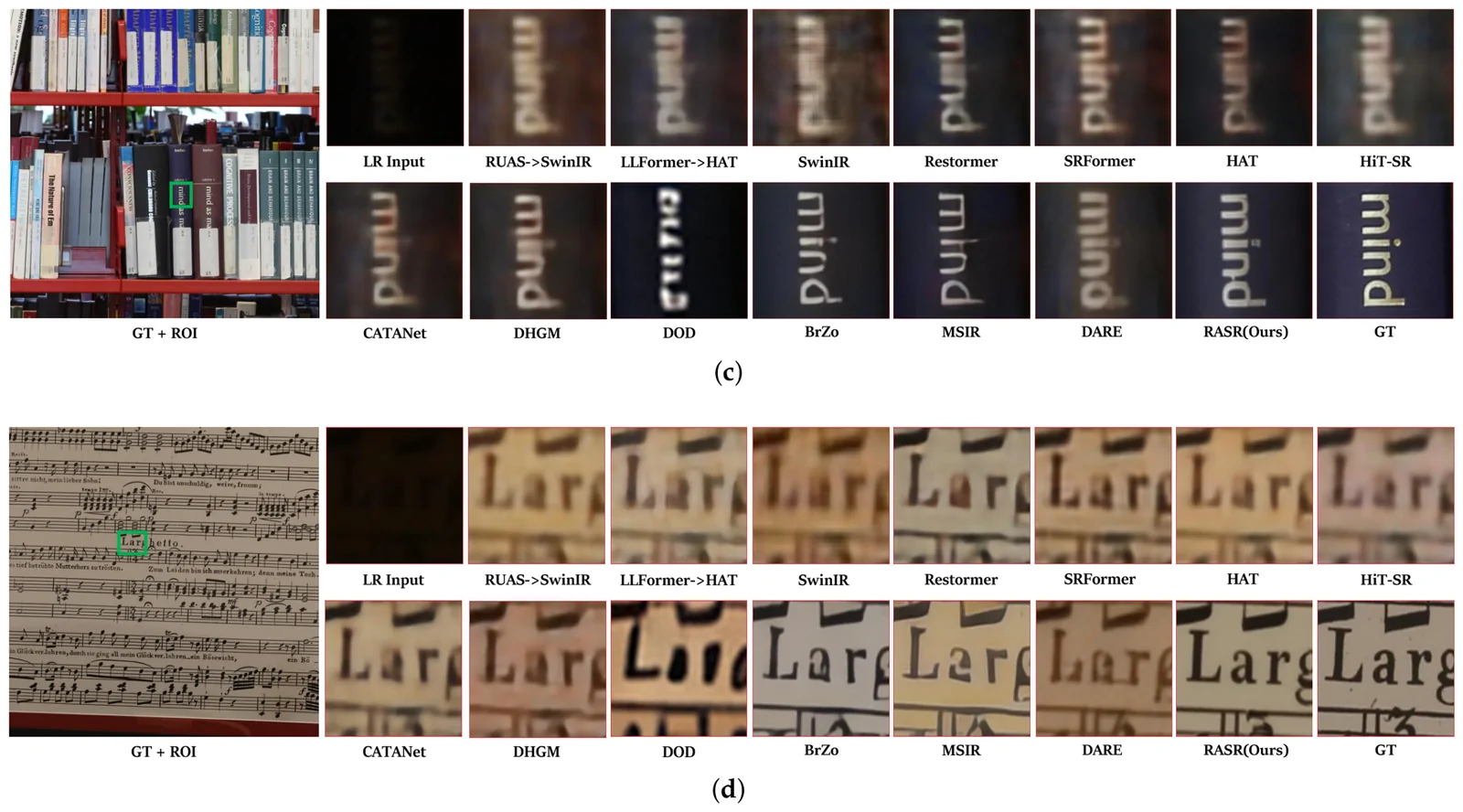
Qualitative comparison on RELLISUR. (**a**,**b**) Two representative samples on the ×2 task. (**c**,**d**) Two representative samples on the ×4 task. From left to right: LR input, compared methods, RASR (Ours), and GT; zoomed regions are shown where applicable.

Figure 3 shows qualitative comparisons on the RELLISUR evaluation set for the ×2 and ×4 tasks. Each row compares the low-resolution input with representative methods, including cascaded pipelines (RUAS → SwinIR, LLFormer → HAT), mainstream super-resolution/restoration models (SRFormer, Restormer, HAT, HiT-SR, SwinIR, CATANet), low-light super-resolution methods (DARE, MSIR, BrZoNet), and cross-task restoration methods retrained on RELLISUR (DHGM for deraining + super-resolution and DOD for all-in-one restoration), together with the ground truth (GT). RASR restores clearer dark-region details, sharper textures, and more natural brightness, with fewer artifacts especially at high-frequency structures such as text and edges.

**Figure 4:**
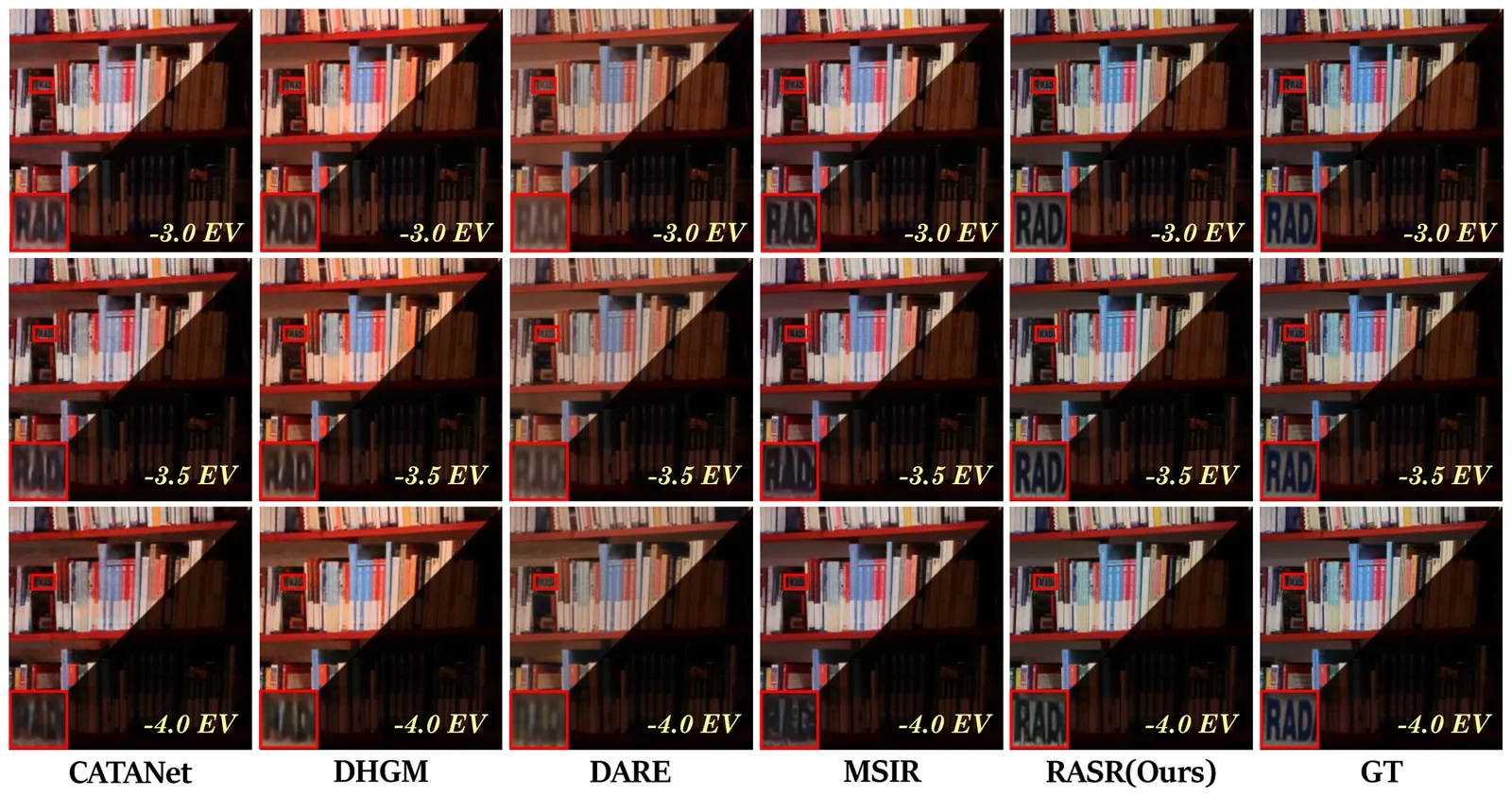
Qualitative comparison at three exposure levels. From top to bottom: −3.0, −3.5, and −4.0 EV. From left to right: CATANet, DHGM, DARE, MSIR, RASR (Ours), and GT. Red boxes indicate the regions enlarged in the insets.

Figure 4 compares restoration results at three exposure levels: −3.0, −3.5, and −4.0 EV. RASR recovers more legible characters in the highlighted bookshelf regions across these levels, including the darkest case shown. This comparison provides a qualitative view of reconstruction under varying exposure conditions.

## 5. Conclusions

In this paper, we propose RASR, a Retinex-guided adaptive one-step diffusion framework for low-light super-resolution. Its core lies in decoupling the illumination and content of a dark image before diffusion, using the reflectance as the content carrier, and predicting a per-sample adaptive timestep via teacher–student distillation, which improves low-light super-resolution reconstruction quality on the RELLISUR benchmark while using a single diffusion integration step per tile. To support stable training, we further design a two-stage loss strategy that first establishes a stable latent and pixel foundation with reconstruction losses, and it then gradually introduces perceptual and adversarial losses to refine textures. Experiments show that RASR achieves competitive performance on the RELLISUR evaluation set: it comprehensively leads existing methods on all three metrics on the ×2 task, and on the ×4 task it leads on PSNR and LPIPS among all compared methods, with SSIM roughly comparable to DHGM [58] (a deraining + super-resolution method retrained on the same RELLISUR training set), with the most significant improvement in perceptual quality (LPIPS); ablation studies further validate the effectiveness of each key design—the two-stage loss consistently outperforms the single-stage loss on both ×2 and ×4 tasks, using the reflectance as the content carrier is overall superior to directly encoding the dark image in both fidelity and perceptual quality, and adaptive timesteps improve all three mean metrics on ×2 and provide a small perceptual benefit on ×4, accompanied by a slight SSIM decrease.

Several cross-domain ideas suggest future extensions. FreDNet’s frequency-sensitive representations [60] motivate alternatives to the Sobel-based teacher measurement; DST’s local–global representations and VBRS’s synchronized optimization across bitrate conditions [61] suggest ways to coordinate reflectance and illumination features and training across degradation conditions. The spatial compensation of CMSC in STSc-GCN [62] motivates exploiting spatial or inter-branch relationships, whereas its temporal MSTC mechanism is relevant to a future video extension. These are prospective extensions to the present RASR framework.

## Figures and Tables

**Table 1:** RASR efficiency on one NVIDIA A800-SXM4 80 GB GPU, BF16, batch size 1. Resolution denotes the model input/output grid; memory is peak allocated memory. Runtime is the mean ± sample standard deviation over ten repeats of one prepared input per resolution.

Task	Resolution	Tiles	Time (s/Image)	Peak Memory (GiB)
×2	1248×1248	4	0.6827±0.0015	7.70
×4	2496×2496	16	3.4495±0.0048	18.41

**Table 2:** Quantitative comparison on the RELLISUR evaluation set (425 images, best results in bold).

Method	Task	×2				×4		
		PSNR↑	SSIM↑	LPIPS↓		PSNR↑	SSIM↑	LPIPS↓
EDSR [44]	Generic SR	18.38	0.679	0.466		17.69	0.679	0.623
D-DBPN [45]	Generic SR	18.70	0.682	0.460		17.96	0.674	0.575
ESRGAN [23]	Generic SR	18.08	0.655	0.300		17.18	0.647	0.471
RDN [46]	Generic SR	18.79	0.701	0.455		18.21	0.701	0.584
RCAN [47]	Generic SR	19.76	0.712	0.426		19.07	0.712	0.550
SRFBN [48]	Generic SR	18.42	0.662	0.510		17.67	0.665	0.640
PAN [49]	Generic SR	18.78	0.693	0.450		18.10	0.700	0.559
MSRResNet [23]	Generic SR	18.15	0.677	0.451		17.59	0.684	0.581
SwinIR [3]	Generic SR	18.39	0.641	0.587		17.51	0.664	0.697
Restormer [50]	Generic SR	21.22	0.728	0.390		20.28	0.721	0.503
SRFormer [51]	Generic SR	19.56	0.705	0.472		18.71	0.706	0.623
HAT [52]	Generic SR	20.22	0.719	0.460		19.73	0.715	0.573
HiT-SR [53]	Generic SR	16.29	0.624	0.590		17.65	0.683	0.643
CATANet [54]	Generic SR	19.81	0.711	0.422		18.89	0.708	0.551
RUAS [55] → SwinIR	Pipeline	17.16	0.617	0.581		17.06	0.662	0.663
LLFormer [56] → HAT	Pipeline	21.22	0.721	0.462		20.12	0.718	0.585
MIRNet [57]	LLIE	21.05	0.720	0.436		19.78	0.704	0.599
BrZoNet [36]	LLSR	22.79	0.745	0.243		21.41	0.726	0.383
DHGM [58]	Derain + SR	22.66	0.763	0.373		20.15	**0.733**	0.544
DOD [59]	AiOIR	20.81	0.720	0.256		17.84	0.622	0.488
MSIR [37]	LLSR	21.36	0.733	0.289		19.75	0.699	0.392
DARE [38]	LLSR	20.25	0.723	0.387		19.39	0.715	0.455
**RASR (two-stage)**	LLSR	**23.70**	**0.771**	**0.202**		**21.45**	0.729	**0.319**

All compared methods are retrained on the same RELLISUR training set used in this work. Task notation: “Generic SR” denotes methods designed for conventional image super-resolution; “Pipeline” denotes cascaded low-light enhancement followed by super-resolution; “LLIE” denotes low-light image enhancement without resolution upscaling; “LLSR” denotes methods dedicated to low-light image super-resolution; “Derain + SR” and “AiOIR” denote deraining + super-resolution and all-in-one image restoration methods, respectively.

**Table 3:** Ablation of two-stage versus single-stage loss (425 images, trained 12,000 steps).

Scale	Strategy	PSNR↑	SSIM↑	LPIPS↓
×2	Single-stage	23.00	0.751	0.212
×2	Two-stage	**23.70**	**0.771**	**0.202**
×4	Single-stage	20.92	0.711	0.348
×4	Two-stage	**21.45**	**0.729**	**0.319**

**Table 4:** Ablation of key components (trained 12,000 steps, best in bold).

Scale	Setting	PSNR↑	SSIM↑	LPIPS↓
×2	Baseline (reflectance + adaptive)	**23.70**	**0.771**	**0.202**
×2	Direct dark-image encoding	23.32	0.758	0.216
×2	Fixed timestep	23.36	0.756	0.211
×4	Baseline (reflectance + adaptive)	**21.45**	0.729	**0.319**
×4	Direct dark-image encoding	20.97	0.729	0.338
×4	Fixed timestep	21.36	**0.731**	0.323

**Figure A1:**
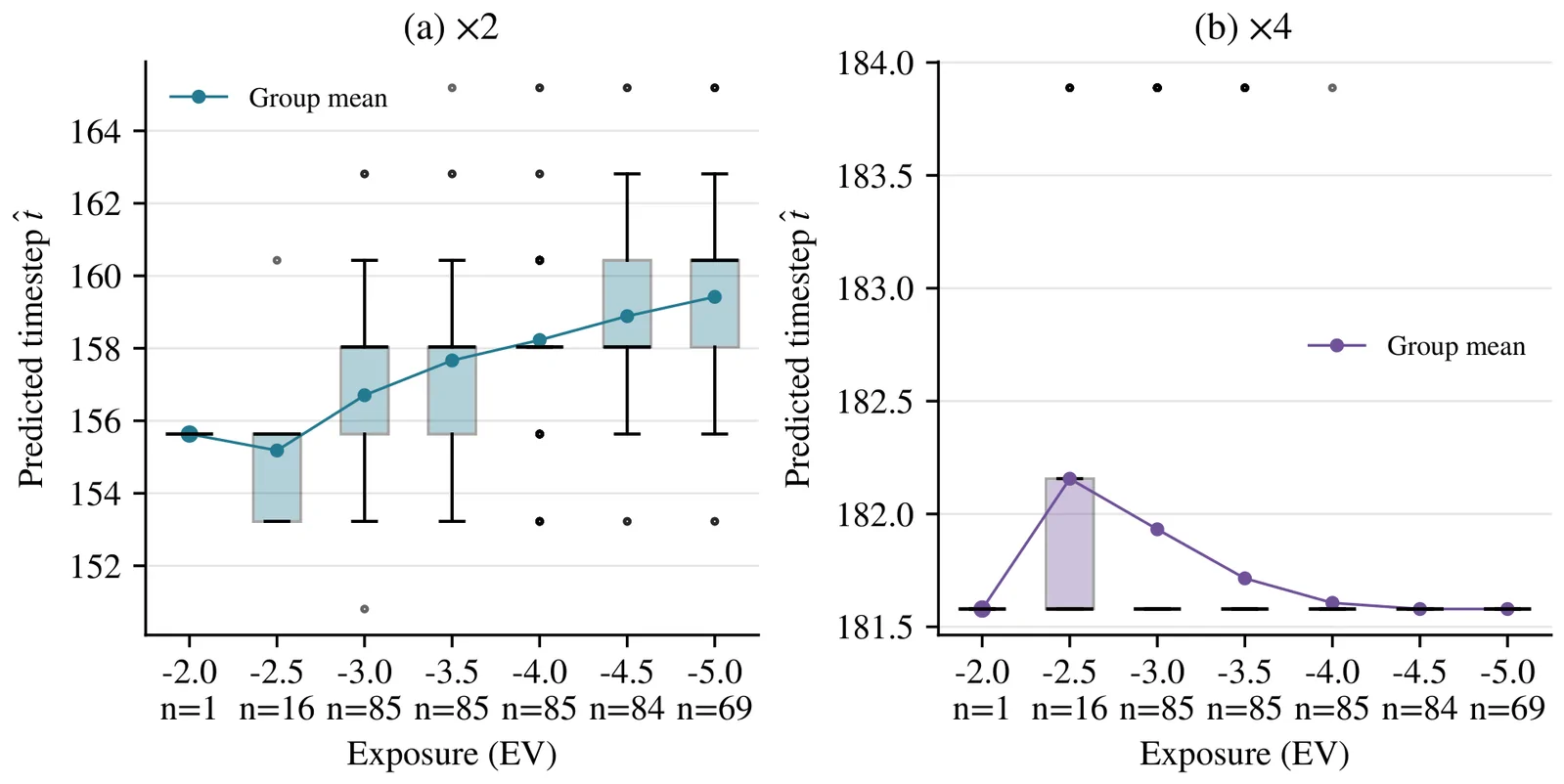
Predicted timesteps grouped by exposure on the RELLISUR test set. Each panel uses its own vertical range. Labels give the number of images in each group. Boxes show the interquartile range, horizontal lines denote medians, whiskers extend to the most extreme observations within 1.5 interquartile ranges, and small dots show outliers. Joined circles indicate group means. The single observation at −2.0 EV is shown as a point.

## Data Availability

The RELLISUR dataset used in this study is publicly available. The code and trained models are available from the authors upon reasonable request.

## Appendix A. Implementation and Evaluation Details

### Appendix A.1. Paired Data and Retinex Initialization

The ×2 and ×4 models are trained separately. Each uses 3610 pairs from Train/LLLR and the corresponding Train/NLHR-Duplicates/X2 or X4 directory. Evaluation uses 425 pairs from Test_crop/LLLR and Test_crop/NLHR-Duplicates/X2 or X4. Images are paired by exact filename, and the training and test LQ filename sets are disjoint. The test set contains 85 scenes with five exposure images per scene. Test preparation retains the top-left 624×624 region of each 625×625 LQ image and the corresponding 1248×1248 or 2496×2496 region of its HQ reference. This removes the bottom and right borders before inference; no additional border is removed when computing the metrics.

The Retinex decomposition branch is separately pretrained on these 3610 training pairs, without external or test data. Its objective is the same decomposition loss used in subsequent joint training: low- and normal-light reconstruction, structure-aware illumination smoothness, illumination-prior constraints, and reflectance consistency. The resulting net_g_141000.pth checkpoint initializes RASR. The decomposition branch is frozen for the first two epochs and unfrozen at the start of the third epoch (zero-based epoch index 2).

Restored outputs are clipped to [0,1] and saved as 8-bit RGB PNGs before evaluation. PSNR uses all color-channel values; SSIM is computed per channel and averaged using channel_axis = 2. Both use scikit-image with data_range = 255, without Y-channel conversion, border cropping, or metric-time color correction. OpenCV reads both images in BGR order, which leaves these channel-aggregated scores unchanged. LPIPS explicitly converts BGR to RGB and normalizes the input to [−1,1]. The reported RASR checkpoints at both scales use all 425 test pairs, with no missing GT images, read failures, or shape mismatches.

### Appendix A.2. LoRA Configuration

The VAE encoder uses rank r=64 and α=64; the SD3 Transformer uses r=128 and α=128. Both adapters therefore have scaling α/r=1, zero dropout, and PEFT Gaussian initialization (init_lora_weights = "gaussian"). The target-module names are:

- **VAE encoder:** conv1, conv2, conv_in, conv_shortcut, conv, conv_out, to_k, to_q, to_v, and to_out.0.
- **SD3 embedding and attention:** x_embedder, attn.to_k, attn.to_q, attn.to_v, attn.to_out.0, attn.add_k_proj, attn.add_q_proj, attn.add_v_proj, and attn.to_add_out.
- **SD3 feed-forward layers:** ff.net.0.proj, ff.net.2, ff_context.net.0.proj, and ff_context.net.2.

The pretrained VAE/Transformer weights remain frozen during adaptation. For inference, the adapters are merged into their corresponding weights; the parameter counts in Appendix A.5 distinguish the pre- and post-merging networks.

### Appendix A.3. Discriminator Heads

The frozen DINOv3-ConvNeXt-Large backbone supplies its first three feature stages, with 192, 384, and 768 channels. Table A1 specifies the three trainable heads for 1024×1024 training crops. Each of the four head blocks consists of zero-padded BlurPool, a spectrally normalized 3×3 convolution with padding 1, and LeakyReLU with slope 0.2. The table lists convolution strides; each preceding BlurPool separately uses stride 2. Its depthwise 4×4 filter is the normalized outer product of [1,3,3,1], with one-pixel zero padding on each side. Each head ends with another BlurPool and a spectrally normalized 1×1 convolution that produces a single logit channel.

**Table A1:** Discriminator-head configurations for 1024×1024 training crops. Channel counts refer to the successive convolution outputs.

Feature Channels	Head Channels	Convolution Strides
192	96, 96, 96, 96	1, 2, 2, 2
384	192, 96, 96, 96	1, 1, 2, 2
768	384, 192, 96, 96	1, 1, 1, 2

For each input, binary cross-entropy with logits is averaged over the spatial positions and batch and summed over the three heads. The smoothed target is 0.8 for real samples and for the generator’s adversarial objective; discriminator fake samples use target 0. Color, translation, and cutout DiffAugment are applied during adversarial training. The backbone remains frozen, while differentiation through its features provides gradients to the generated image for the generator update.

### Appendix A.4. DINOv3-Based DISTS-Style Loss

This training loss adapts the structure/texture similarity construction of DISTS [39] to frozen DINOv3-ConvNeXt-Large features [40], with fixed channel weights. The loss implementation receives the restored image and reference normalized to [−1,1] by N. For either normalized input *x*, define the RGB feature level $f_{0}\left(x\right)=\left(x+1\right)/2$. The deep-feature input is normalized using ImageNet mean (0.485,0.456,0.406) and standard deviation (0.229,0.224,0.225). It then passes through the first three backbone stages. Channel-wise L2 pooling follows each stage, and the pooled output is passed to the next stage as well as retained for the loss. Thus, the four feature levels have channel counts $\left(C_{0},C_{1},C_{2},C_{3}\right)=\left(3,192,384,768\right)$.

The L2-pooling kernel is obtained by removing the zero endpoints of a five-point Hann window and normalizing the outer product of the remaining three values. Equivalently, for $h={\left[1,2,1\right]}^{⊤}$,

(A1) $$G=\frac{hh^{⊤}}{16},\;P{\left(u\right)}_{c}=\sqrt{G*u_{c}^{2}+10^{-12}},$$

where convolution is channel-wise, with stride 1 and zero padding 1. The RGB level is not pooled or ImageNet-normalized for loss computation.

For a paired image and reference, let $x_{lcj}$ and $y_{lcj}$ denote values at spatial position *j* in channel *c* of feature level *l*, whose spatial size is $N_{l}$. For each sample, compute

(A2) $$\begin{array}{cccc} \mu_{x} & =\frac{1}{N_{l}}\sum_{j}x_{lcj}, & \mu_{y} & =\frac{1}{N_{l}}\sum_{j}y_{lcj},\\ v_{x} & =\frac{1}{N_{l}}\sum_{j}{\left(x_{lcj}-\mu_{x}\right)}^{2}, & v_{y} & =\frac{1}{N_{l}}\sum_{j}{\left(y_{lcj}-\mu_{y}\right)}^{2},\\ q_{xy} & =\frac{1}{N_{l}}\sum_{j}x_{lcj}y_{lcj}-\mu_{x}\mu_{y}. & & \end{array}$$

The mean and centered-feature similarity terms are

(A3) $$S_{lc}=\frac{2\mu_{x}\mu_{y}+c_{1}}{\mu_{x}^{2}+\mu_{y}^{2}+c_{1}},\;T_{lc}=\frac{2q_{xy}+c_{2}}{v_{x}+v_{y}+c_{2}},\;c_{1}=c_{2}=10^{-6}.$$

For a batch of *B* pairs, the loss is

(A4) $$L_{\mathrm{DINO}\text{-}\mathrm{DISTS}}=\frac{1}{B}\sum_{b=1}^{B}\left[1-\frac{1}{2\sum_{l=0}^{3}C_{l}}\sum_{l=0}^{3}\sum_{c=1}^{C_{l}}\left(S_{lc}^{\left(b\right)}+T_{lc}^{\left(b\right)}\right)\right].$$

All similarity-term weights are fixed at 1/[2(3+192+384+768)]. Backbone parameters remain frozen, while gradients through feature computation optimize the restored image. The evaluation metric is AlexNet LPIPS; this modified DINOv3-based loss is used only during training.

### Appendix A.5. Efficiency Measurement and Parameter Accounting

Inference measurements use one NVIDIA A800-SXM4 GPU with 80 GB memory, BF16 precision, and batch size 1. For each scale, the timing script selects the first PNG in filename order from the saved evaluation-output directory, converts it to RGB in [0,1], and multiplies its intensities by 0.35. Its dimensions match the model-input/output grid for that scale: 1248×1248 for ×2 and 2496×2496 for ×4. These are prepared model inputs, rather than the raw LQ image dimensions.

Each prepared image undergoes two warm-up passes and ten timed passes, with CUDA synchronization. Runtime is reported as the mean and sample standard deviation of those ten repetitions (Table A2). The measurements characterize latency at benchmark-matched resolutions; neither the mean nor its standard deviation describes variation across the 425 test images.

**Table A2:** Inference resource measurements. Runtime is mean ± sample standard deviation over ten repeated passes of one prepared image per scale. Memory values are in GiB.

Scale	Resolution	Time (s)	Allocated	Reserved
×2	1248×1248	0.6827±0.0015	7.70	10.26
×4	2496×2496	3.4495±0.0048	18.41	28.21

Timing covers the complete restoration forward pass: Retinex decomposition, adaptive-timestep prediction, VAE encoding, tiled SD3 velocity prediction and one-step integration, and VAE decoding. It excludes model loading, text-embedding computation, image I/O, input preparation and transfer to the GPU, and output conversion and saving. Peak allocated memory measures PyTorch 2.13.0 tensor use; peak reserved memory also includes memory retained by the caching allocator. These are inference measurements on the tested system, not estimates of training memory or minimum deployment GPU capacity.

The restoration network contains approximately 2.291 billion parameters before LoRA merging and 2,118,061,928 parameters (approximately 2.118 billion) after merging. It comprises the SD3 Transformer, VAE, Retinex decomposition network, and adaptive-timestep module; text encoders used to precompute prompt embeddings and training-only loss/discriminator networks are excluded. After Retinex unfreezing, the restoration branch has approximately 179.33 million trainable parameters: SD3/VAE LoRA adapters, 173.42 M; Retinex, 1.86 M; and the adaptive-timestep module, 4.05 M. The discriminator heads add approximately 4.98 M trainable parameters, giving 184.31 M across both branches. Their DINOv3 backbone remains frozen.

The measured configuration uses 128×128 latent tiles, corresponding to 1024×1024 image pixels, with nominal overlap of 64 latent pixels (512 image pixels). The last tile is aligned to the image boundary where necessary. The two resolutions require 4 and 16 tiles, respectively. Each tile uses one SD3 Transformer evaluation and contributes to one Flow Matching integration step; whole-image computation therefore depends on resolution and tile count. Retinex processing and VAE encoding/decoding are included in the measured whole-image runtime.

### Appendix A.6. Adaptive-Timestep Diagnostics

The diagnostics use the 12000-step checkpoints evaluated in Table 4, with frozen weights, on all 425 test pairs (85 scenes, five exposure images per scene). The fixed-timestep configuration uses zero-based scheduler index 700, corresponding to t≈564.13 and σ≈0.5641 on the 1000-point SD3 schedule. These values refer to the scheduler grid before the inference implementation casts them to BF16 (t=564, σ=0.5625).

For the student–teacher comparison, the LQ input follows the original inference preprocessing and Retinex decomposition. The resulting reflectance and illumination maps are bilinearly resized to a maximum side length of 512 before the FP32 student predicts its continuous noise level. The reflectance and paired HQ reference are encoded by the checkpoint-adapted VAE encoder and the frozen original SD3 VAE encoder, respectively, after normalization to [−1,1]. Retinex processing and VAE encoding use BF16. Following the training formulation, one posterior sample is drawn per encoder and image (HQ first, then LQ); the random seed is 123+i for zero-based sorted image index *i*. The teacher is constructed under no_grad. Errors compare continuous $\hat{\sigma }$ with $\sigma_{T}$, and nearest-grid timesteps $\hat{t}$ with $t_{T}$, before the final BF16 cast. For either quantity *q*, MAE is $N^{-1}\sum_{i}\left|{\hat{q}}_{i}-q_{T,i}\right|$ and RMSE is ${\left[N^{-1}\sum_{i}{\left({\hat{q}}_{i}-q_{T,i}\right)}^{2}\right]}^{1/2}$, with N=425.

**Table A3:** Held-out student–teacher prediction errors on all 425 test pairs.

Scale	σ MAE	σ RMSE	*t* MAE	*t* RMSE
×2	0.0259	0.0371	26.01	37.09
×4	0.0266	0.0363	26.66	36.43

Figure A1 groups predictions by the exposure values encoded in the official test filenames. The numbers of observations are unequal; the −3.0, −3.5, and −4.0 EV groups each contain the same 85 scenes. Other exposure groups contain subsets of these scenes, so their marginal distributions also reflect different scene compositions. For the same 85 scenes at −3.0, −3.5, and −4.0 EV, the mean predicted timesteps are 156.71, 157.67, 158.23 on ×2 and 181.93, 181.71, 181.61 on ×4, respectively. The ×2 distributions show a small upward shift as exposure decreases. The ×4 response is limited: 402 of 425 predictions map to t≈181.58, and the remaining 23 map to t≈183.89; continuous predictions span $\hat{\sigma }\in \left[0.1805,0.1838\right]$.
